# Supplementary figures and images for: A light-entrained clock mechanism in a hydrozoan jellyfish synchronizes evening gamete release
Source: PLoS Biol. 2026 Jan 6;24(1):e3003502. doi: 10.1371/journal.pbio.3003502 (PMC12773804; doi:10.1371/journal.pbio.3003502)

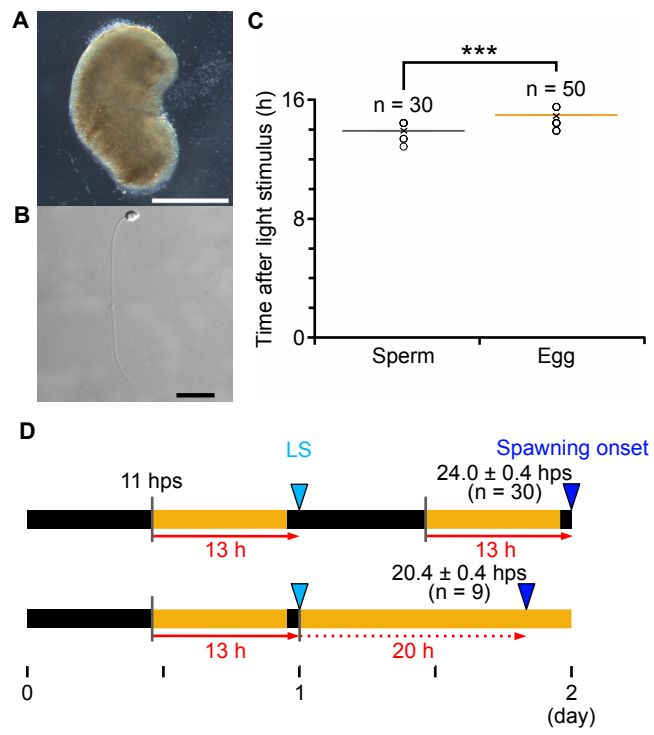

Supplement: S1 Fig — (A) Isolated male gonads that release sperm. Scale bar: 0.5 mm. (B) A high magnification image of a sperm. Scale bar: 10 µm. (C) Comparison of male and female spawning time measured from the onset of light stimuli. Sperm release occurred earlier (13.0 ± 0.3 hours) than egg spawning (13.9 ± 0.3 hours). Sperm release timing is defined by its onset from a jellyfish, as it continues up to 1 hour. Bars indicate standard deviation; n = number of jellyfish. (Welch’s test, *** p ≤ 0.001) (D) Sperm release also exhibits regular spawning at dusk under a light–dark cycle and autonomously under constant light. Egg spawning and sperm release observation data are available at https://doi.org/10.1101/2025.05.05.651927. (PDF) [file pbio.3003502.s001.pdf]

**A**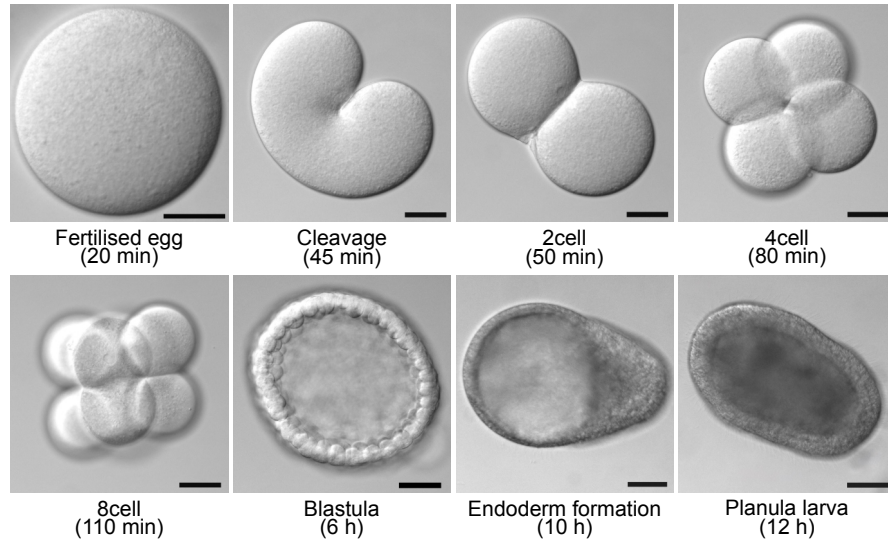**B**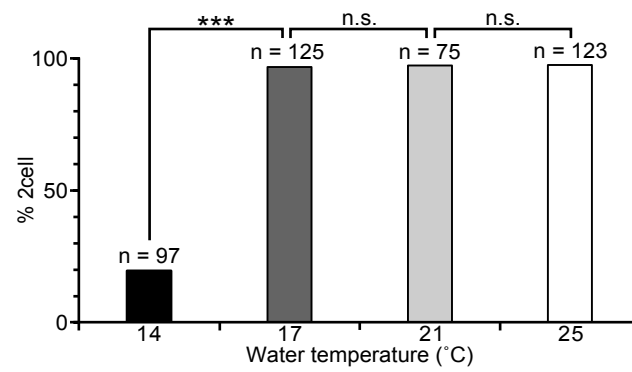

Supplement: S3 Fig — (A) DIC images of each embryonic stage and planula larva. Scale bars: 50 µm. Time after fertilization is indicated in parentheses. (B) Fertilization efficiency at different temperatures (chi-squared test, *** p ≤ 0.001, n.s. p ≤ 0.05). Fertilization efficiency data are available at https://doi.org/10.1101/2025.05.05.651927. (PDF) [file pbio.3003502.s003.pdf]
