# Supplementary material for: A light-entrained clock mechanism in a hydrozoan jellyfish synchronizes evening gamete release
Source: PLoS Biol. 2026 Jan 6;24(1):e3003502. doi: 10.1371/journal.pbio.3003502 (PMC12773804; doi:10.1371/journal.pbio.3003502)
Supplement: S2 Fig — (A) DIC images of oocytes and their nuclei at different stages. The staging criteria were based on Amiel and colleagues [26] for Clytia hemisphaerica, with additional clarification that late stage II is defined by peripheral migration of GV and presence of nucleoli, while stage III is determined by the disappearance of nucleoli. Arrowheads indicate nucleoli. Bars are 50 µm for oocyte panels and 10 µm for nucleus panels. (B) Characteristics of oocyte stages and their occurrence after the previous spawning. (PDF) [file pbio.3003502.s002.pdf]

A

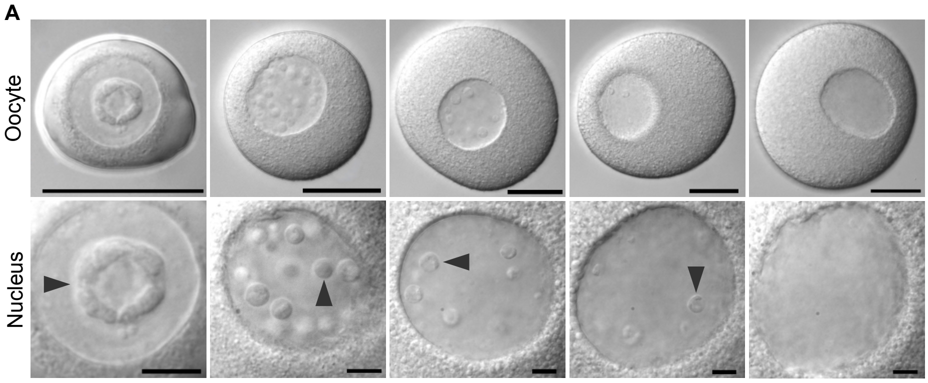

B

Oocyte stage	Diameter (μm)	Position of GV	Nucleolus		Response to WPRPa	Observable period (hps)
			Number	Size		
Stage I	< 50	Centre	Single	Large	No	0–24
Stage II early	< 100	Centre	> 30	Small	No	0–24
Stage II mid	< 150	Off–centre	10–20	Small	No	10–16
Stage II late	160–180	Peripheral	3–10	Small	No	16–18
Stage III	160–180	Peripheral	No		○	18–22
