# Supplementary material for: A light-entrained clock mechanism in a hydrozoan jellyfish synchronizes evening gamete release
Source: PLoS Biol. 2026 Jan 6;24(1):e3003502. doi: 10.1371/journal.pbio.3003502 (PMC12773804; doi:10.1371/journal.pbio.3003502)
Supplement: Attachment — Submitted filename: Responses-to-reviwers_2025-10-03.docx [file pbio.3003502.s011.docx]

Reviewer #1: Review: "A light-modulated clock mechanism in a hydrozoan jellyfish that synchronises evening gamete release." (PBIOLOGY-D-25-01890R1).

The manuscript by Kitsui and colleagues describes a novel rhythmic phenomenon relevant for the biology of hydrozoan jellyfish; but more generally, it is an interesting report for understanding clocks.

In many marine animals with external fertilisation, the timing of gametes release is a life-or-death choice. The survival of the species depends on the ability of eggs and sperms to find each other in the vastity of the sea, and without getting confused with others. Light is an obvious synchroniser. Indeed, light-dark changes, either because of the sun or of the moon cycle, are universal reference points that all individuals can recognise and align to. Moreover, by setting release to occur at a specific phase of the cycle, different species can occupy a unique temporal niche in an otherwise overlapping space, just like booking a table at the restaurant. In the hydrozoan jellyfish Clytia hemisphaerica, a light stimulus triggers the secretion of a maturation-inducing hormone (MIH) from the gonads, which drives the (final) meiotic maturation and the release of the oocytes. Perhaps not surprisingly, the MIH-secreting cells express opsins. In this manuscript the authors describe a novel species, Clytia sp. IZ-D, a close relative of C. hemisphaerica. Interestingly, in this new species, oocytes are released not as a direct response to a light stimulus but according to what it seems an autonomous clock. It is the first time such a phenomenon is described in hydrozoan. Note, hydrozoan have lost key components of the animal circadian clock (the transcription regulators Clock and Cyc/Bmal1). The manuscript then takes us through a series of well-thought experiments to demonstrate that the rhythm is endogenous (it is not driven by an external rhythmic stimulus) and to some extent is self-sustained, although light-dependent. The rhythm is entrainable by a light-dark cycle (although the limits and the strength of entrainment are quite fuzzy) but it is not temperature compensated (the period of the rhythm changes extensively with temperature). Thus, we have a rhythmic process that is no longer an 'hourglass' (in C. hemispherica rhythmicity would terminate without the daily resetting by light) but it is not a fully-fledged circadian clock (self-sustained, entrainable and temperature compensated) either. Physiological experiments then show how such a clock could emerge from the hourglass mechanism. In C. hemisphaerica light triggers the release of MIH at a time when the oocytes are ready to respond to it. In C. sp. IZ-D, the oocytes acquire that competence more slowly, requiring, under light conditions (light-dark or constant light), at least 18h from the previous spawning. Thus, the addition of a delay separating two interacting components: production/release of MIH and oocytes development (ultimately resulting in their MIH-responsiveness), bend into a cycle an otherwise linear process (oocytes development).

In its essence a circadian clock is composed by an effector process (producing the output) and a modulatory process. What makes of two linear processes a cycle is the timing of their interaction. There must be a period when the two processes cannot interact (delay) and that interval must be determined by additional independent processes (cooperativity). The best-known clocks are based on transcription translation feedback loops (with such an organisation), but it is the organisation, not the actual elements, what makes the clock.

However, in C. sp. IZ-D, light is required to synchronise the development of the oocytes and temperature has a large effect on their maturation timing. Thus, additional control elements, which could contribute 'cooperativity', are not independent, explaining why such a clock does not graduate as a fully-fledged circadian clock.

In summary, the manuscript describes an interesting phenomenon that goes well beyond the immediate curiosity of hydrozoan enthusiasts. It is well written and is built on a progression of technically simple (albeit laborious) but carefully thought through experiments. Far too often we are 'dazzled into submission' by technical tour-de-force of the latest omics, parading brute 'funding' power rather than ingenuity. Here it is the opposite, and it is refreshing!

We greatly appreciate these insightful comments. As well as providing us with valuable input to improve the manuscript, the referee gives an excellent overview for the general reader, emphasising the importance of this work in the context of their expertise in evolutionary chronobiology. We were also happy to read that our patient investigation unveiling unexplored marvels of nature has been appreciated. We hope that this work will inspire marine animal enthusiasts in the wider public, not only amongst researchers.

Reviewer #2: Kitsui and colleagues describe a new species of Clytia and its interesting dark spawning pattern, which contrasts with morning spawning in the more widely used Clytia hemisphaerica. I found this manuscript to be quite fascinating, carefully done and well communicated. The experiments are robust, and the quantifications seem appropriate. The schematics throughout the paper make it easier to follow. The core finding is a dual influence of light exposure on oocyte growth and the acquisition of its ability to respond to meiotic inducing hormone, resulting in a consistent spawning event 14 hours after the previous dark to light transition. These conclusions are reached by a clever series of dark/light manipulations, ovary explant and oocyte isolations, in vitro treatments with the maturation inducing hormone, and light microscopy. I have only minor suggestions and support publication but do suggest one experiment that could help with the interpretations, if it is feasible to do so.

Specifically, I am left wondering: how does the dark-light transition stall/delay MRC acquisition? What is the step that is regulated by light exposure? The authors conclude that MRC is acquired "autonomously" within oocytes by extracting oocytes at 17hps under a L12/D12 cycle. But I would be quite surprised if the oocytes themselves are detecting light (through an opsin?) and delaying their acquisition of MRC (by repression of a receptor? Something else?). Could delay of MRC instead require some signal from ovarian somatic cells to the oocyte? For the experiments in Fig. 6, oocytes received the dark to light stimulus several hours before they were removed from the gonad - time for such a signal to occur. Could the authors perform an experiment as in Figure 6, but by extracting oocytes immediately upon light exposure? Under these conditions, would the oocytes acquire MRC faster than if they remained in the ovary longer after light exposure?

Thank you for your constructive suggestion. Based on this, we performed oocyte isolation experiments with different timing (now added to Figs. 6 and 7, which have been reorganised). We, however, found that oocytes could not reach stage III when they were isolated earlier than 16 hps. This suggests that, besides programming oocytes based on the light conditions, gonad somatic cells are simply required for correct oocyte development at earlier growth stages. We have added this finding to the results section. Although we thus cannot currently answer the question raised by the reviewer, we consider his/her comment extremely insightful. In the long term, we hope to establish a new approach to distinguish MRC acquisition programming from oocyte development. While it would be highly challenging to devise an in vitro oocyte culture system from earlier stages, it may be possible to use *C. hemisphaerica* gonad as an incubator. For example, oocytes may acquire MRC immediately upon light stimulation if stage I/II oocytes from Clytia sp. IZ-D were transplanted to the *C. hemisphaerica* gonad.

Minor Point:

Page 14: References to Suppl. Fig. 1 appear to be incorrect - should they be Suppl. Fig 2?

Thanks. Corrected.

Reviewer #3: This article was a "tour de force" in the elucidation of a clock mechanism synchronizing gamete release in a new species of hydrozoan jellyfish. I found the work to be clearly explained, comprehensive, and well executed. I have only very minor comments and essentially feel that the manuscript is ready for publication. I very much enjoyed reading this paper.

My main comment is that I am uncomfortable with the way the authors use the term "zeitgeber" to refer to a light-dark regime (p 7, and throughout). The term is more commonly used to refer to the stimulus like "light." I would prefer if the authors replace "zeitgeber" with "light-dark cycle" or "light regime" in most places. Alternatively, on p26, they refer to a "zeitgeber cycle." I haven't seen that usage before, but it seems reasonable.

Thank you for the input on the use of the word "Zeitgeber". We were, in fact, unsure of the use of this term and do not want to insist on keeping it. We removed “Zeitgeber” throughout.

It might be worth including an explicit discussion of temperature compensation somewhere in the manuscript. Some of the described processes are clearly temperature-sensitive (e.g, p 8). It would be helpful to provide the context that temperature compensation is a canonical feature of "true" circadian rhythms. Along these lines, the authors use the term "quasi circadian" (p 12), which could benefit from some definition or discussion.

Thank you for this suggestion. It is a very interesting point and indeed we didn’t discuss the temperature-dependency. Brief discussions were added in p. 10 (Results) and 21 (Discussion).

I found the second paragraph of the section "MIH-induced maturation is conserved" to be a little unsatisfying. [as an aside: p 19: though BMAL/CLOCK are absent, other conserved transcription factors may play a similar role (e.g., PAR-bZip family)...this might be mentioned (optional)]. The authors talk about how there may not be a novel clock mechanism, suggest that hormones may be gradually released, but never really resolved the question as to how this would create such precise timing. I think it would be ok if a sentence were added to indicate that this aspect is still unresolved/mysterious.

We took this comment very seriously. Indeed, we have argued amongst ourselves over months about the mysteries of the molecular mechanisms. A section of the Discussion was extensively rewritten concerning this point. While BMAL1/CLOCK are missing the well-conserved CRY1 orthologue in *Clytia hemisphaerica and clearly could be involved in spawning regulation, as we now mention*

Other small suggestions:

p 3 "MIH comprises...." (should this be "is comprised of"?)

Corrected.

p 5 It's a little clear what the authors mean in the last line but "circadian clock-independent" Perhaps they mean not regulated by clock/cycle, but they have clearly demonstrated an autonomous cycle with a ~daily free-running rhythm. Sentence could use minor re-wording.

We totally agree. These were inconsistent within a few sentence distances. We have re-worded this part.

p 12 a small syntax issue in the last line. "were isolated...and tested their response..."

Agreed and corrected.

p 17: consider replacing "adjusted" with "entrained"

True. Entrained is more accurate in this context. We corrected it.

p 24...here authors mention an "autonomous circadian cycle"...but in other places, they discuss clock-independent (p 5...though maybe this is meant to be CLOCK-independent) and "quasi circadian"...if they do want to say circadian here, there is the caveat that it doesn't seem to be temperature compensated.

Yes. Our writing was confusing because we mixed up authentic circadian clock (transcriptional feedback oscillator) mechanisms employing BMAL1/CLOCK and CRY/PER, which is likely missing in hydrozoan, and autonomous daily spawning cycle, shown in this work. We have now made these distinctions clear.
